# Dynamic Aging: Channeled Through Microenvironment

**DOI:** 10.3389/fphys.2021.702276

**Published:** 2021-07-21

**Authors:** Qing Tan, Na Liang, Xiaoqian Zhang, Jun Li

**Affiliations:** State Key Laboratory of Medical Molecular Biology, Department of Biochemistry and Molecular Biology, Institute of Basic Medical Sciences, Chinese Academy of Medical Sciences and Peking Union Medical College, Beijing, China

**Keywords:** aging, SASP, microenvironment, NAD^+^, intercellular communication

## Abstract

Aging process is a complicated process that involves deteriorated performance at multiple levels from cellular dysfunction to organ degeneration. For many years research has been focused on how aging changes things within cell. However, new findings suggest that microenvironments, circulating factors or inter-tissue communications could also play important roles in the dynamic progression of aging. These out-of-cell mechanisms pass on the signals from the damaged aging cells to other healthy cells or tissues to promote systematic aging phenotypes. This review discusses the mechanisms of how senescence and their secretome, NAD^+^ metabolism or circulating factors change microenvironments to regulate systematic aging, as well as the potential therapeutic strategies based on these findings for anti-aging interventions.

## Introduction

With 100% incidence rate and individualized symptoms, aging is a highly complex process which simultaneously affects multiple organ systems (Ahadi et al., 2020). In addition to probing for the cell-autonomous mechanisms of aging, there is growing awareness of that deregulated intercellular communication contributes to decline in tissue/organ health with aging (Lopez-Otin et al., 2013). Intercellular communication refers to both direct interactions between neighboring cells and indirect cell communication via various message signals. A harmonious intercellular communication system is very important for organ development, stress response, cell survival and etc. In contrast, a disordered intercellular communication can be detrimental in aging progression and promote aging-related diseases (Fafian-Labora and O’Loghlen, 2020).

The best-known means of regulating intercellular communication are soluble factors in blood or extracellular matrix that can easily cross cell membrane to take effect through autocrine or paracrine signaling (Acosta et al., 2013). These soluble factors exist in various forms including proteins, metabolites or nucleic acids. Protein factors are the most well-studied, with the proactive secretome of senescent cells, known as senescence-associated secretory phenotype (SASP) factors, accounting for the vast majority. Senescent cells amass with age and secret more and more SASP factors into extracellular matrix, resulting in chronic low-grade immune response activation, or “Inflammaging,” which systematically compromise physiological functions and contribute to age-related dysfunctions in different organs or tissues such as neurodegeneration (Frederiksen et al., 2019; Ogrodnik et al., 2021), atherosclerosis (Childs et al., 2016), osteoarthritis cancer (Jeon et al., 2018), and kidney dysfunction (Valentijn et al., 2018), etc. Apart from SASP factors, non-SASP circulating protein factors and metabolites, particularly NAD^+^, have been extensively researched in recent years for their roles in aging development. Studies designed to counteract the change of these factors or metabolites caused by aging have shown that they are promising targets for anti-aging interventions. Moreover, recent studies have accumulated evidence that non-coding RNA molecules are linked to several biological aspects of aging such as senescence or autophagy, indicating more research attention needed.

Here, we review some major means of intercellular communication that affect aging such as senescence and senescence associated secretion phenotype (SASP) in the context of physiological or pathological scenario. We also go over the regulatory mechanisms of circulating aging-related factors including proteins factors, NAD^+^ and non-coding RNAs, as well as potential anti-aging strategies that target them. In light of the close tie of NAD^+^ metabolism with aging, we discuss how different organs use this highly mobile cofactor to shape cellular microenvironment. In light of the close tie of NAD^+^ metabolism with aging, we-discuss how different organs use this highly mobile cofactor to shape cellular microenvironment.

## Part 1: Cellular Senescence and Sasp Modulate the Development of Aging Microenvironment

Hayflick and Moorhead (1961) observed that the primary cells would have replicative exhaustion after about 50 generations *in vitro*. It was the first evident that replicative senescence was the driver of organismal aging. Cellular senescence is a relatively stable physiological state in which cells lose their ability to proliferate and resistant to apoptosis. It is characterized by permanent cell cycle arrest, enlarged cell size, persistent DNA damage response, abnormal protein degradation, impaired nutrient response, and increased inflammation level (Lopez-Otin et al., 2013). Senescent cells have been proven to accumulate in the aged mice tissues, and clearance of senescent cells can ameliorate these aging phenotypes (Baker et al., 2011, 2016). Hereby, these causative connections qualify cellular senescence as a hallmark of aging and attract enormous research interest (Lopez-Otin et al., 2013). Although senescent cells are almost found in almost all kinds of tissues of aged animals, it is believed that senescence originates from single damaged cells and could propagate to neighboring or remote tissues via its SASP secretome (Coppe et al., 2010; Childs et al., 2015). A strategy aiming to remove senescent cells named Senolysis has shown great potential in retarding aging phenotypes in multiple aging-related diseases including osteoarthritis, atherosclerosis (Childs et al., 2016), chemotoxicity (Baar et al., 2017), chronic obstructive pulmonary disease (COPD) (Chilosi et al., 2013), and idiopathic pulmonary fibrosis (IPF) (Cai et al., 2020). It is not exaggerated that senescence and SASP are the major players when it comes to the cellular communication between tissues and microenvironmental homeostasis during aging. In this part, by summarizing the features of senescence, we discuss about the cell types mostly vulnerable to senescence in different tissues and how senescent cells jeopardize functions of other tissues via SASP secretion.

### The Features of Senescence and SASP

**FIGURE 1:**
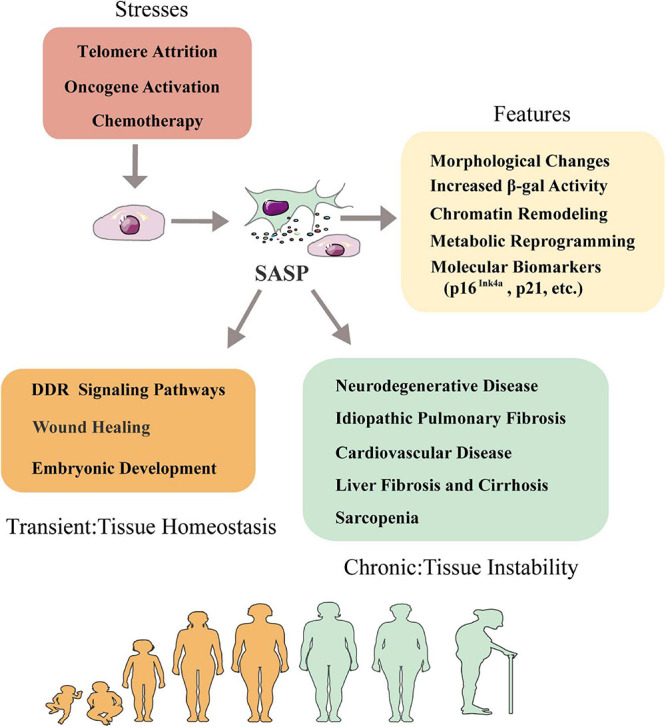
Senescence-associated secretory phenotype and tissue homeostasis. Senescence is triggered by telomere attrition, oncogene activation and radiation or chemotherapy treatment. Senescent cells can be characterized by a series of features including morphological changes, increased β-galactosidase activity, chromatin remodeling, metabolic reprogramming, secretion of SASP factors and molecular biomarkers like p16^Ink4a^, p21, etc. Influence of SASP in tissue homeostasis is context dependent: in short term, SASP factors involve DDR signaling pathways, wound healing and embryogenesis to help maintain tissue homeostasis; in long term, SASP factors promote immunosenescence in aging-relative diseases such as neurodegenerative diseases, idiopathic pulmonary fibrosis, cardiovascular disease, liver fibrosis and cirrhosis, sarcopenia and skin aging. DDR, DNA damage response.

A number of features of senescence have been characterized, which could be used as proper biomarkers or potential therapeutic targets. Senescent cells generally display dramatically morphological changes, increased β-galactosidase activity, stable cell cycle arrest, persistent DNA damage response, metabolic reprogramming and significant chromatin remodeling (Figure 1). Senescence can be identified by certain molecular biomarkers such as induction of cell cycle inhibitors p21 or p16^Ink4a^, upregulated DNA damage markers γH2AX or ATM, nuclear depletion of HGMB1 and reduced Lamin B1 level (Freund et al., 2012; Tchkonia et al., 2013; Wiley et al., 2016; Kumar et al., 2020). Senescent cells can release of a special panel of SASP secretome. The secretion of SASP factors change microenvironment and affect even remote tissue via paracrine, which is believed to contribute to organ degeneration with aging (Campisi, 2013).

The phenotypic manifestations of SASP are heterogeneous and induced by different internal and external stimulus including telomere attrition, DNA damage, oncogenic activation, mitochondrial dysfunction or epigenetic alterations (Lopez-Otin et al., 2013; Tchkonia et al., 2013). The SASP factors are mainly made of different types of soluble components including pro-inflammatory cytokines, growth factors, chemokines and extracellular matrix-degrading proteins (Gorgoulis et al., 2019). This particular combination of signaling factors and the proteases that degrade extracellular matrix (ECM) to facilitate signal transduction has made SASP a powerful mechanism to modulate intercellular communication. The secretion of SASP factors is considered as a major detrimental aspect of senescence because it promotes chronic inflammation, induces fibrosis and causes stem cell exhaustion (Freund et al., 2010; He and Sharpless, 2017; Schafer et al., 2017). However, it has also been shown to favor embryonic development or wound healing, suggesting whether beneficial or detrimental effects the SASP exerts depends on the physiological and pathological context (Wilkinson and Hardman, 2020). For example, a recent study has shown that transiently exposing the primary mouse keratinocytes to SASP factors increased cell stemness and regenerative capacity *in vivo*, while prolonged exposure caused secondary senescence and hindered regeneration (Ritschka et al., 2017). This suggests senescence has more complicated physiological roles than currently understood.

**FIGURE 2:**
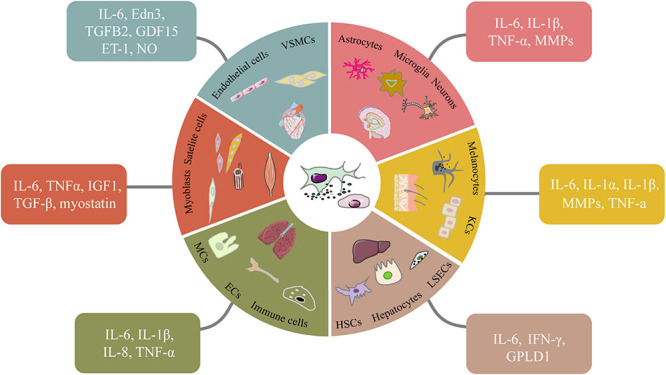
Senescent cell types and the SASP factors they secret in different tissues. In different tissues, various senescent cells secrete classic or specific SASP factors in autocrine and paracrine ways. Some signaling molecules such as NO which accordingly change to elevate chronic inflammation, induce tissue dysfunction and accelerate aging. VSMCs, vascular smooth muscle cells. LSECs, liver sinusoidal endothelial cells; HSCs, hepatic stellate cells.

The relationship between SASP and microenvironmental homeostasis is complex and paradoxical (Figure 1). In some cases, SASP factors can help to maintain tissue homeostasis by positively regulating tissue regeneration and repair (Nehme et al., 2020). For instance, transient secretion of platelet-derived growth factor AA (PDGF-AA), a SASP factor categorized as growth factor, attracts leukocytes to the wounded sites and contributes to wound resolution (Demaria et al., 2014). In other cases, SASP factors like IL-6, IL-8, MCPs and MIPs can act on neighboring cells to disrupt tissue homeostasis, cause chronic inflammation and accelerate disease exacerbation in the body (Terlecki-Zaniewicz et al., 2018). Therefore, the effects of SASP on microenvironment could be highly individualized given the fact that different tissue or organ has different cell compositions and mitotic status. Next, we discuss the effects of SASP factors on different tissues in the following subtopics (Figure 2).

### The Effects of SASP Factors on Immune Functions

The effects of aging on immune system can be summarized as two terms: “inflammaging” and “immunosenescence.” “Inflammaging” refers to the chronic low-level pro-inflammatory development within tissues during aging (Franceschi et al., 2018). Immunosenescence demonstrates as a gradual reduction in immune cells, inmate and adaptive immune functions with aging (Aw et al., 2007b). Despite their conceptional difference, inflammaging and immunosenescence join forces with SASP to drive age-related immune function decline, forming a vicious cycle. Inflammaging increases the production of inflammatory factors, most of which are SASP factors, aggravates the reduction in immune response and eventually leads to immunosenescence. The decreased immune response impedes immune cells clearance of damaged cells, increasing the risk of generating more senescent cells and SASP factors, which lead to a higher level of inflammaging. Therefore, Senescence and SASP factors have the most comprehensive effects on immune system which might indirectly drive the function decline of other systems.

#### Immunosenescence Compromises Immune Responses

In the age-associated diseases such as neurodegeneration, cardiovascular diseases or cancer, SASP factors are considered to be the major reason for immunosenescence mechanism to cause persistent prolonged inflammation and progenitor cell exhaustion (Franceschi and Campisi, 2014; Jian et al., 2020). Immunosenescence decreases the response to vaccinations, impairs wound healing and enhance the susceptibility to virus infection, malignancy, or autoimmunity (Aw et al., 2007a; Sanada et al., 2018). SASP factors are temporally regulated, the first wave of SASP factors such as transforming growth factor-β1 (TGF-β1) and TGF-β3 are typically immunosuppressive, whereby the second wave of the SASP often consists of proinflammatory factors (Ito et al., 2017). One of the most important functions of immune system is immunosurveillance on tumorigenesis. SASP secretion may change immune system into pro-tumorigenesis environment by releasing pro-inflammatory factors in autocrine and paracrine ways. Senescent fibroblasts secrete a large number of CXC-chemokine ligand 1 (CXCL1), CXCL2, IL-6, insulin-like growth factor binding proteins (IGFBPs) and colony stimulating factors (CSFs) to accelerate the development of tumors (Nacarelli et al., 2019). The IL-1 pathway significantly promoted pancreatic tumor progression and immune cell infiltration by controlling the secretion of IL-1α and other SASP factors (Lau et al., 2019). Together, these factors enhance the severity of cancer and accelerate age-related cell damage, thus further promote a metabolic microenvironment suitable for cancer development. However, in some cases senescence could be used against tumorigenesis. A combination of IFN-γ and tumor necrosis factor (TNF) directly drove tumor cells into senescence (Braumuller et al., 2013). Another exciting study used chimeric antigen receptor (CAR) T cells that target an antigen (uPAR) specific to senescent human cells to reverse aging-associated pathologies and improve outcome of cancer treatment (Amor et al., 2020). Hereby, senescence has made immune system out to be a double-blade sword on cancer, which might have potential in designing cancer-treating drugs.

#### Inflammaging Promotes Inflammatory Microenvironment

Various causes contribute to inflammaging which include SASP (Lopez-Otin et al., 2013). Acute inflammation, essential for keeping body healthy, occurs immediately after stimulated by environmental or internal stressors such as alcohol, trauma, or pathogens (Han et al., 2015). Opposite to acute inflammation, there is another form of inflammation characterized as chronic, sterile, low-grade inflammation aggravating with aging process, named “Inflammaging” (Franceschi et al., 2018). As an important driving force of aging and age-associated diseases, Inflammaging is associated with dysregulated immune system and increased secretion of pro-inflammatory factors such as IL-1, IL-6, IL-8, IL-13, IL-18, TNF, etc. (Franceschi et al., 2018; Fulop et al., 2019). IL-6, a major cytokine contributing inflammaging, is considered to be a useful biomarker in the elderly people to predict 3-year to 6-year mortality (Alley et al., 2007). Gradually increased inflammatory states in elderly people result in decreased physical functions and increased susceptibility to chronic diseases (Vasto et al., 2009). However, the centenarian and their offspring often have relatively lower inflammation index (Arai et al., 2015). Inducing chronic inflammation by knocking out *nfkb1*, a subunit of NF-κB, accelerated aging phenotypes in multiple organs (Jurk et al., 2014). Besides age, there are other risk factors of inflammaging such as visceral obesity (Frasca et al., 2017), genetic variants (Smith and Humphries, 2009), gut dysbios (Rampelli et al., 2013), accumulation of senescent cells (van Deursen, 2014), and mental stress (Miller and Raison, 2016). The pro-inflammatory SASP cytokines secreted by senescent cells are the major carriers responsible for the spread and aggravation of inflammaging which turns microenvironment into hostile conditions.

Inflammaging also contributes to the development of many age-associated chronic diseases such as cardiovascular disorders, neurodegenerations, cancers and metabolic diseases (Ferrucci and Fabbri, 2018; Armutcu, 2019). The oxidized low-density lipoprotein on artery walls caused initiation and development of atherosclerosis by inducing inflammation, while the atherosclerotic plaques could aggravate inflammation (Libby, 2012; Libby and Kobold, 2019). Inflammation mediated by microglia activation significantly promote neurodegeneration in the AD brain (Webers et al., 2020). Indomethacin, an anti-inflammatory agent, reduces systemic inflammation and deposition of AA amyloid in mice brain and attenuates the progression of Alzheimer’s disease (Guo et al., 2002). Circulating inflammatory cytokines IL-1β, IL-6, and CRP were elevated in T2 diabetes patients via nuclear factor-κB (NF-κB) and JUN N-terminal kinase (JNK) dependent pathways. IKKβ is a key downstream mediator of tissue inflammation. Using salicylates to inhibit IKKβ ameliorate glucose tolerance and decrease insulin resistance in the patients with type 2 diabetes (Hundal et al., 2002). Gut dysbiosis featured by changed gut microbiota composition and increased mucosal barrier permeability occurs with aging, it is reported to promote inflammaging and trigger cancers (Biragyn and Ferrucci, 2018). Interestingly, inflammasome can protect against carcinogenesis by activating innate immune reactions and triggering the pyroptosis of premalignant cells (Zitvogel et al., 2012, 2017). This suggest inflammaging might not simply play a vicious function, it is also a compensation mechanism to fight infections or tumorigenesis in response to immune function decline due to immunosenescence.

The mechanisms underneath inflammaging and its contribution to age-related diseases are poorly understood, yet in a great need to clarify. Inflammaging participates in several cellular and molecular mechanisms such as pro-inflammatory cytokine secretion, inflammasome activation, cellular senescence, dysregulation of the protein homeostasis, DNA damage response, etc (Ferrucci and Fabbri, 2018). Inflammaging is accompanied by increased pro-inflammatory or chemotactic factors such as TNF-α, IL-1β, IL-6, IL-8, IL-18, and C-C motif chemokine 2 (CCL2) (Vandanmagsar et al., 2011; Shakeri et al., 2018). These cytokines, most of which are SASP members, further activate several inflammatory signal pathways (Schmitz et al., 2011; Liu et al., 2017). For example, cigarette can activate pulmonary NF-κB pathway, leading to symptoms of IPF in mice. SRT1720, an activator of SIRT1, can suppress NF-κB pathway to improve lung health (Rahman et al., 2012). Vitamin D3 also inhibits inflammation via JAK-STAT3 pathway, another important inflammatory pathway, and attenuates the development of neurological diseases (Yasukawa et al., 2012; Boontanrart et al., 2016). Inflammasomes are assembled under the stimulation of damage-associated molecular patterns (DAMP) and pathogen associated molecular patterns (PAMPs). Activation of inflammasomes elevated the IL-1β and IL-18 levels and induced pancreatic β-cell apoptosis (Strowig et al., 2012). The mechanisms of inflammaging are complex involving pathways both in immunity regulation and aging progression. A better understanding of these unknown pathways could help to develop methods to suppress inflammaging and improve physiological functions.

The interventions extending lifespan or improving healthspan in animal models usually can suppress inflammation. Calorie restriction, the most classic way to extend lifespan, can effectively attenuate pro-inflammatory cells and inflammatory factors (Ma et al., 2020). Clearance of p16^Ink4a^-positive senescent cells in INK-ATTAC mice via injection of senolytic drug AP20187 alleviates inflammation, delays oncogenesis and extends lifespan (Baker et al., 2016). Increasing eosinophils, an anti-infection white blood cell, reduces inflammation in adipose tissue and partially improves muscle tissues in old mouse (Brigger et al., 2020). Aspirin, a widely used anti-inflammatory drug, reduces beta-galactosidase activity and increases telomerase activity in replicative senescence endothelial cells (Bode-Boger et al., 2005; Zelenay et al., 2015). Overexpression of SIRT7 in breast cancer cells prevents the metastasis into lung by inhibiting TGF-β signal pathway (Tang et al., 2017). Therefore, modulating inflammation could be effective therapeutic targets on increasing longevity and preventing age-related diseases.

### The Effects of SASP Factors on Neurological Functions

Neurodegeneration is a common aging-related pathology. Senescence has been widely found in most types of cells in aged brain including neurons, microglia, astrocytes and neural stem cells (Si et al., 2021). In the frontal cortices of Alzheimer’s disease patients, high levels of p16^Ink4a^, γH2AX, and MMP1 were found in astrocytes linking to reduced lactic acid and antioxidant (Bhat et al., 2012). Senescent neural stem cells disrupt the generation of neurons, while the senescent neurons can induce secondary senescence on other cellular types causing functional decline. In aged mice, microglia exhibit increased production of the proinflammatory cytokines IL-6, IL-1β, and TNF-α, which damages the brain and increases neurodegeneration (Frederiksen et al., 2019). In old rats, astrocytes, microglia, and neurons secret pro-inflammatory cytokines including TNF-α and IL-1β to induce progressive degeneration (Lana et al., 2016). Senescent endothelial cells could promote the rupture of the blood-brain barrier, which makes it easier for the peripheral immune cells to enter the brain (Maciel-Barón et al., 2018). Senescence in brain can be aggravated by pathological changes. Aggregation of tau protein induced cellular senescence in the brain. Tau transgenic mice exerted senescence-like phenotypes including DNA damage, mitochondrial dysfunction and SASP (Musi et al., 2018). Overall, senescence is one of the major causes for the postmitotic brain cells to lose proper functions. The SASP factors generated from cells within or outside of central nervous system disrupt tissue homeostasis, increase neuroinflammation, and eventually contribute to the initiation or the progression of neurodegenerative diseases (Figure 2).

### The Effects of SASP Factors on Respiratory Functions

With age advancing, the senescent epithelial cells and fibroblasts accumulate in adult lungs (Chen et al., 2019). The aging phenotypes in lung include pulmonary inflammation, high susceptibility to infections, loss of lung elastic recoil and enlargement of the distal air space (MacNee, 2016). The main causes of senescence in lung are replicative senescence, radiation or chemotherapy, and cigarette smoking (Rashid et al., 2018). In aging process or chronic lung diseases, endothelial cells and particularly fibroblasts undergo senescence (Yanai et al., 2015; Qiu et al., 2019; Gea et al., 2020). During the pathogenesis of chronic lung diseases especially IPF or COPD, cellular senescence and SASP factors act as pivotal driving forces. The senescent epithelial cells in the lungs of COPD patient produce SASP factors IL-1β, IL-6, IL-8, and TNF-α (Kumar et al., 2014), which is usually due to NF-kB pathway activation in a p38-dependent manner (Maciel-Baron et al., 2016). Many intrinsic or external stressors can trigger SASP secretion via NF-κB signaling such as DNA damage, oxidative stress, inflammation, lung injuries, etc. (Rovillain et al., 2011; Salminen et al., 2012; Dong et al., 2015). The SASP response is activated by p21^CIP1^, followed by activation of p38 mitogen-activated protein kinase and Janus-activated kinases, eventually results in the activation of NF-κB and secretion of proinflammatory cytokines (Soto-Gamez and Demaria, 2017). These SASP cytokines alter microenvironment adversely resulting in elevated inflammation, lung fibrosis and onset of aging and diseases. The senescence phenotypes in the fibroblasts of pulmonary fibrosis are so typical that many senolytic drug candidates or strategies are tested on mouse model of IPF (Lehmann et al., 2017; Schafer et al., 2017; Cai et al., 2020), and a recent progress is a human clinical trial testing therapeutic effects of dasatinib and quercetin (DQ) in human IPF patients (Justice et al., 2019).

### The Effects of SASP Factors on Cardiovascular Functions

The heart is one of the least regenerative organs in the body and highly susceptible to cardiovascular diseases. Studies have shown that aging leads to structural and functional changes in myocardial cells. In the old people (>70 years old), more than 50% cardiac progenitor cells (Sca-1^+^/c-kit^+^/CD31^–^/CD45^–^/Tryptase^–^) showed higher senescent biomarkers including p16^Ink4a^ induction, SA-b-gal positivity, and increased γH2AX level and SASP secretion (Lewis-McDougall et al., 2019). Single-nucleus quantification and clustering analysis showed that in heart cardiomyocyte and endothelial cells are the major cell types (Wolfien et al., 2020). Senescent cardiomyocytes can secrete atypical SASP factors such as endothelin 3 (EDN3), transforming growth factor β 2 (TGFB2) and growth differentiation factor 15 (GDF15) to induce senescence in neighboring cells (Anderson et al., 2019; Li et al., 2020a). endothelial cells form the lining of blood vessels and serve as the interface between vessels and tissues (Daneman and Prat, 2015). Senescent endothelial cells show increased endothelin-1 (ET-1) release and reduced nitric oxide (NO) production, which elevate local inflammation, induce atherosclerosis and damage vascular integrity (Uryga and Bennett, 2016). Vascular smooth muscle cells (VSMCs) are the basic component of the medial layer of arteries responsible for contraction/dilation (Lacolley et al., 2012). Apart from classic senescence features, senescent VSMCs affected signaling pathways like NO/cGMP signaling, voltage-dependent and Ca^2+^-activated ion channels and the interactions of VSMCs with ECM (Rubio-Ruiz et al., 2014). Senescent VSMCs downregulated collagen and upregulated multiple cytokines in an IL-1α–dependent manner, driving adjacent VSMCs and ECs into a proinflammatory state (Gardner et al., 2015). Senescent VSMCs have abnormal response to Angiotensin-II (Ang-II), which causes increased DNA methyltransferase expression, elevated blood pressure and collagen deposition, ultimately lead to cardiac fibrosis (Domenighetti et al., 2005; Bacanamwo et al., 2007). Together, senescent heart cells and SASP cytokines contribute to the pathological symptoms of aged heart including arterial stiffening, cardiomyocyte hypertrophy and elevated myocardial fibrosis (Lakatta, 2003; Lakatta and Levy, 2003).

### The Effects of SASP Factors on Liver and Metabolism

The liver is essential for regulating protein biosynthesis, energy metabolism and detoxification (Hunt et al., 2019). With aging, the number of hepatocytes decreases along with increased polyploidy, accumulation of senescent cells and reduced mitochondrial oxidative capacity (Basso et al., 1998). The hepatocytes can be induced into senescent state and subsequently produce SASP factors. Senescence and SASP can significantly affect metabolism and secretion panel of liver cells. The metabolism of the fibroblasts under influence of SASP factors undergo dramatic changes from mitochondrial dysfunction, hydrogen peroxide production, to a transition toward aerobic glycolysis (Pazolli et al., 2012), which in turn lead to increased production of high-energy metabolites into the microenvironment, along with ROS (Fane and Weeraratna, 2020). In a liver fibrosis and cirrhosis model, p53-expressing senescent stellate cells release IFN-γ and IL-6, promote macrophage activation toward tumor-inhibiting M1-state, whereas P53 null cells secrete IL-3, IL-4, and IL-5 that hinder macrophage activation and trapped in tumor-promoting M2-state (Lujambio et al., 2013). As a secretion organ, liver can secret its own anti-aging protein factor named Glycosyl-phosphatidylinositol-specific phospholipase D1 (GPLD1). It is carried to brain by blood to improve brain cognition function and neuron growth in rodents, while senescence in hepatocytes could impair this function (Horowitz et al., 2020). In summary, liver constantly guard metabolism and endocrine factors for inter-tissue communication that are both susceptible to senescence and SASP proinflammatory factors. Interestingly, in contrast to the common scenario of avoiding senescence and SASP, some research attempt to induce senescence on hepatocellular carcinoma cells by small molecules. A recent study successfully used the inhibitor of DNA-replication kinase CDC7 to selectively induce senescence in liver cancer cells with TP53 gene mutation but showing no effect on normal cells (Wang et al., 2019). It suggests senescence is not only a “destroyer,” but also could be a useful weapon against cancer.

### The Effects of SASP Factors on Muscle Functions

Normally, aging phenotypes in muscle include weight loss, lower grip strength, slow gait speed and decreased exercise endurance. Cellular senescence drives muscle aging mainly by causing muscle stem cell dysfunction. In muscles of old mice satellite cells entering senescence state due to high expression of p16^Ink4a^ lost their regeneration ability, which can be reversed by silencing p16^Ink4a^ (Sousa-Victor et al., 2014). Muscle fusion and regeneration require successful completion of cellular communications mediated by lipids, membrane proteins, exosomes and cytokines such as Insulin-like growth factor (IGF-1), TGFβ or myostatin (Demonbreun and McNally, 2017). However, the main components of SASP factors like IL-6 and TNF-α counteract insulin signaling and erythropoietin cascades, leading to insulin resistance and sarcopenia, a typical muscle aging phenotype (Beyer et al., 2012). It is interesting to notice that transient expression of IL-6 could be favorable for muscle regeneration after injury or during *in vivo* reprogramming (Ocampo et al., 2016; Chiche et al., 2017), indicating a context-dependent effect of IL-6. The spread of SASP cytokines also change muscle into more inflammatory microenvironment, like it does to other tissues. This age-elevated inflammation can promote muscle fibrosis and lead to chronic myopathy. For example, Sarcopenia has a strong inflammatory pathology due to senescence in muscle (Dalle et al., 2017). In contrast, strong evidence proves that exercise can reduce inflammation and improve muscle functions (Churchward-Venne et al., 2012). Overall, senescence and SASP appear to be the major contributors to muscle aging which validates elimination of senescent cells would be the most effective strategy to rejuvenate muscle functions.

### The Effects of SASP Factors on Skin

Skin, as the largest organ of the human body, is the first protective shield against the external environment. Aging skin in nature is characterized by a loss of epidermal and dermal thickness, accompanied by loss of elasticity, dryness, wrinkle formation, dyspigmentation, slower wound healing, decreased collagen, and susceptibility to cancer (Ma et al., 2001; Mazini et al., 2021). Ultraviolet (UV) irradiation, a great risk factor of skin aging, induce DNA damage, ROS production, protein oxidation and lipid peroxidation by promoting the expression of MMPs (Noonan et al., 2012). MMP-1 is the primary cause of wrinkle formation for breaking down collagen fibers type I and III (Dong et al., 2008). Keratinocytes (KCs) is the outermost layer of the skin. UV induces senescence in KCs, resulting in increased SA-β-gal activity, decreased Lamin B1 expression and elevated secretion of TNF-a, IL-1a, IL-1b, or IL-6 (Wang and Dreesen, 2018). Similarly, senescent melanocytes secrete IL-6 and IL-8, which accelerate skin aging via activation of the TLR4-mediated ERK pathway (Seo et al., 2018; Victorelli et al., 2019). Activation of P38 MAPK signaling also increases the expression of MMPs that break down collagen to promote skin aging (Mavrogonatou et al., 2018). Senescence usually inhibits non-cell-autonomous growth and considered to suppress tumorigenesis. However, new evidence found that cellular senescence could stimulate skin carcinogenesis by elevating p38MAPK and MAPK/ERK signaling (Alimirah et al., 2020). Whether this is a common feature of senescence or a special event in skin requires further investigation.

## Part 2: Circulating Factors That Influence Aging Microenvironment and Possible Therapeutic Targets

Circulating factors are the most fundamental intercellular regulators of microenvironment for their easy access into all the tissues or organs, and for the same reason, they are the most appealing therapeutic targets for anti-aging intervention. The metabolites and proteome profile in human blood have shown profound changes with aging (Auro et al., 2014; Lehallier et al., 2019). Understanding how these changes influence the whole system and drive the process of aging is crucial to developing efficient drugs for anti-aging medications. In the following section, we discuss three types of circulating factors that have seen substantial recent research advance that have been made such as NAD^+^, circulating proteins and RNA molecules, as well as their mechanisms and therapeutic potential.

### NAD^+^ Is a Major Regulator of Intercellular Communication and a Promising Therapeutic Target of Anti-aging

Nicotinamide adenine dinucleotide (NAD^+^), a coenzyme for redox reactions and cofactor for NAD^+^-dependent enzymes represented by sirtuin family, CD38 and poly (ADP-ribose) polymerases family, is well known for its involvement in energy metabolism, DNA repair, epigenetic regulation, immune response, and cell senescence (Covarrubias et al., 2021). There is an age-dependent decline of NAD^+^ content in various human tissues, which is linked to many aging-related diseases including metabolic dysfunctions, cardiovascular dysfunctions, skeletal muscle diseases, neurodegenerative disorders, hearing loss, innate immune dysfunctions, dysrhythmia, retinal degeneration, etc. (Fang et al., 2016; Chini et al., 2017). To counteract the NAD^+^ decline, a new anti-aging strategy to augment NAD^+^ level in the old mice by administrating its precursors NMN or NR have demonstrated wide impacts on improving biological functions from metabolic functions (Elhassan et al., 2019), cognitive conditions (Tarantini et al., 2019), cardiovascular fitness (Das et al., 2018; Kiss et al., 2019a), immune response (Minhas et al., 2019), locomotor activity (Das et al., 2018), to even lifespan (Zhang et al., 2016). The large scale of NAD^+^ impacts is ascribed to its mobile feature which enables it and its derived molecules to circulate in the microenvironment. Increasing NAD^+^ level has a profound influence in the microenvironment and intercellular communication. Here, we discuss NAD^+^’s impact effects into three major aspects: (1) senescence and SASP; (2) impact on major biological processes such as metabolism, immune response, nervous and immune system and muscle function; and (3) functions of extracellular NAD^+^.

#### Interplay of NAD^+^ and Senescence

The general NAD^+^ decline during aging accelerates cell senescence. Hyperactivate PARP1, a NAD^+^-consuming enzyme, leads to decreased NAD^+^ content and induce neuronal loss and elevated cellular senescence in tissues (Fang et al., 2016). Reduced availability of NAD^+^ promoted cellular senescence in aging retinal pigment epithelium (Jadeja et al., 2018). Consistently, repletion of NAD^+^ or reduce the consume of NAD^+^ can rejuvenate senescence cells. For example, increasing NAD^+^ by supplementing its precursors can dramatically rejuvenate senescent cells, including stem cells, immune cells, generative cells, etc. (Han et al., 2016; Zhang et al., 2016; Yoshino et al., 2018). In the other direction, senescence can aggravate NAD^+^ decline which possibly cause more senescence, forming a vicious cycle. When exposed to X-rays or γ-IR, HUVECs and MEFs were induced into senescence and secreted high level of inflammatory SASP factors such as IL-6, IL-8, and MCP-1, causing strong increase in expression and enzymatic activity of CD38, an NAD^+^ catabolizing enzyme in murine bone marrow-derived macrophages (BMDM). Hyperactive CD38 consumed large amount of NAD^+^ which is believed to be one of major causes for NAD^+^ decline with age (Chini et al., 2019). However, inhibiting CD38 reversed age-related NAD^+^ decline and prevented cellular senescence in tissues (Tarragó et al., 2018). Currently, NAD^+^-consuming enzymes inhibitors and NAD^+^ precursors supplementation named as “NAD^+^ boosting” therapy are under extensive study for preventing cell senescence and tissue aging. Interestingly, it is recently found that the intracellular NAD^+^ level in the senescent proliferative exhaustion (PEsen) fibroblasts was somehow relatively maintained (James et al., 2016), despite of the evidences indicating the expression of NAD^+^-consuming enzymes SIRTs, PARPs, CD38 and bone-marrow stromal cell antigen 1 (BST1, CD157) NAD^+^ hydrolases are all dramatically up-regulated in senescent cells (Yamamoto-Katayama et al., 2002; Aksoy et al., 2006; Liu et al., 2009; Mouchiroud et al., 2013; Gerdts et al., 2015). This conflict suggests NAD^+^ metabolism might act in an unconventional way in the senescent cells which require further investigation.

NAD^+^ influences not only cellular senescence but also SASP. NAD^+^ affects SASP secretion mainly via its metabolic-control function. High levels of NAD^+^ enhances glycolysis and mitochondrial respiration, which activates p38MAP kinase and stimulates NF-κB transcription to induce the expression of proinflammatory cytokines (Mendelsohn and Larrick, 2019; Nacarelli et al., 2019). These cytokines elevate inflammatory environment and accelerate cancer progression. In pancreatic cancer mouse model or pancreatic ductal adenocarcinoma (PDAC) mouse model these cytokines elevate inflammatory environment and accelerate cancer progression (Guerra et al., 2011; Nacarelli et al., 2019). NAMPT, a critical NAD^+^ synthesis enzyme, is found up-regulated in many solid tumors supporting tumor cells for their high demand of NAD^+^, which makes it a prognosis index for tumor malignancy or progression (Navas and Carnero, 2021). Inhibiting NAMPT’s by FK866 improved immune microenvironment and the curative effect of chemotherapy (Taniguchi and Karin, 2018; Nacarelli et al., 2019). These evidences remind us that although senescence is tumor-suppressive, effects of SASP factors seem to be tumorigenic. The anti-aging effects of NAD^+^ supplement require a tumor-free microenvironment. In addition to health status of the subject, the timing and dosage of NAD^+^ supplement need to be carefully monitored too. Human body is too complicated for NAD^+^’s effects to be simply positive or negative, and most of time it is somewhere in-between. Therefore, much more still need to be known about how to bring out the “goodness” of NAD^+^ safely in the future.

#### NAD^+^ Regulates Metabolism by Stabilizing Glucose Homeostasis

NAD^+^ can alternate microenvironment by regulating metabolism. For example, NAD^+^ is critical for beta cell function and regulation of glucose homeostasis. In mouse model, defects in NAD^+^ biosynthesis impaired glucose-stimulated insulin secretion in pancreatic islets and disrupted glucose homeostasis, while treatment with NMN could increase glucose-stimulated insulin secretion and improve glucose intolerance (Revollo et al., 2007). Supplement of NMN can also increase insulin concentration in plasma and ameliorate insulin sensitivities of diet- or age-induced diabetes in mice (Yoshino et al., 2011). In a freshly released study of testing NMN in human, Samuel Klein’s team conducted double-blind clinical trial and found that NMN could improve insulin sensitivity, insulin signaling and muscle tissue remodeling in the prediabetic women with overweight or obesity (Yoshino et al., 2021). The reduced NAD^+^ regeneration in mitochondria due to insufficient ATP production drives cell metabolism into aerobic glycolysis, which may change the entire cellular microenvironment (Luengo et al., 2021). The nature of being a widely demanded substrate enables NAD^+^ to have a profound impact in metabolism since many energy-generating reactions require it. It partly explains why supplementation of NAD^+^ could have wide range of anti-aging effects across tissues.

#### NAD^+^ Improves Survival of Immune Cells and Regulates Inflammation Signaling in the Immune System

The immune response function declines to a great extent with age (Weinberger, 2017), whereas NAD^+^ replenishment is reported to ameliorate immune responses (Singhal and Cheng, 2019). NAD^+^ has a natural link to immunology because it serves as substrate of receptor protein CD38/CD157 that are located on cell surface and responsible for evoking immune response. Since it is extracellular NAD^+^ that CD38 mainly utilizes, we will elaborate on it in the later discussion. Here we focus on the link between intracellular NAD^+^ and immune functions. Macrophages play an important role in the resolution of inflammation by secreting various cytokines (Watanabe et al., 2019). Increasing *de novo* biosynthesis of NAD^+^ in aged macrophages can restore oxidative metabolism, improve immune responses and remodel macrophage polarization by activating SIRT3 and increasing QPRT expression (Minhas et al., 2019). NAD^+^ depletion induced by NAMPT inhibitors changes immune microenvironment by recruiting CD3^+^, CD4^+^, and CD8^+^ T cells, and decreasing M2-polarized immunosuppressive macrophages and leads to enhanced checkpoint immunotherapy in glioblastoma (Li et al., 2020b). In addition to macrophages, NAD^+^ also regulate other immune cells. In 2016, a study found that long-term administration of NMN can also significantly improve immune system function by increasing lymphocytes number (Mills et al., 2016). Increasing NAD^+^ level by supplementing NMN can prevent NF-κB activation and control the ongoing inflammatory state by stabilizing telomeres and positively affecting functions of immune cells such as T lymphocytes, B lymphocytes, granulocytes, monocytes, and NK cell population (Omran and Almaliki, 2020). Taken together, efficient NAD^+^ is a crucial for proper immune function and supplementing NAD^+^ has potential to combat the immune dysfunction caused by aging.

#### NAD^+^ Boosters Have Neuroprotective Functions

The decreased NAD^+^ with age compromises nervous system function by causing mitochondrial dysfunction, oxidative damage accumulation, lysosome dysfunction, calcium dysregulation, impaired stress responses, enhanced inflammation, damaged neurogenesis and telomere shortening (Lautrup et al., 2019; Covarrubias et al., 2021). NAD^+^ augmentation by NMN can decrease inflammation, restore mitochondrial function and bioenergetics of nervous system by enhancing neuronal survival, increasing neurovascular coupling responses and cognitive function in aged animals (Lautrup et al., 2019; Poddar et al., 2019). Additional studies suggested that the underlying mechanism might also involve the reduced DNA damage due to increased PARP1 activity and activation of SIRT1-PGC-1a pathways, given that NAD^+^ is a substrate for both PARP1 and SIRT1 (Tarantini et al., 2019). Besides using precursors, overexpressing or activating key enzymes of NAD^+^ biosynthesis to increase NAD^+^ was also proved to be neuroprotective. For example, in *Drosophila*, overexpressing NMNAT could protect against tau or spinocerebellar ataxia 1 (SCA1) induced neurodegeneration (Ali et al., 2012). P7C3, a novel NAMPT activator, can protect against mitochondrial dissolution, enhance hippocampal neurogenesis and ameliorate cognitive in mice, suggesting that it could be a potential therapeutic candidate for treating neurological diseases (Wang et al., 2016). The studies on boosting NAD^+^ in nervous system are relatively more comprehensive than other systems and some remarkable progress has been made in recent years. However, apart from that NAM has been proved to cross blood-brain barrier (Spector and Johanson, 2007), there is no evidence yet demonstrating either NMN or NR can do the same. Therefore, further investigation is needed to dissect whether these improved neurological functions are a direct result of NAD^+^ boosting in the brain or an indirect benefit of the systematic NAD^+^ restoration.

#### NAD^+^ Preserves Muscle Functions by Improving Mitochondrial Biogenesis and Reducing Inflammation

Skeletal muscle, an important organ that performs supportive, sportive, metabolic and endocrine functions, has decreased NAD^+^ content with age due to the down-regulated expression of nicotinamide phosphoribosyltransferase (NAMPT), the rate-limiting enzyme in NAD^+^ biosynthesis (de Guia et al., 2019). Decreased NAD^+^ content may result in loss of skeletal muscle mass and weakened muscle function, a typical muscle pathology named sarcopenia (Dodds et al., 2015), while supplement of NAD^+^ can improve skeletal muscle functions in old mice. For example, specifically overexpressing NAMPT in the skeletal muscle of 24-month-old mice can prominently improve their exercise capacity by increasing glycolytic and TCA cycle flux to levels comparable to young mice (Frederick et al., 2016). Besides genetic approach, replenishing NMN, NR or nicotinamide (NAM), precursors of NAD^+^, can also improve the pathologies of sarcopenia. A 12-month NMN administration to C57BL/6N mice beginning at age of 5 months had effectively increased NAD^+^ content in skeletal muscle, enhanced mitochondrial respiratory capacity, and delayed the aging process based on the global gene expression profiles (Mills et al., 2016). In the obese mice, NMN treatment can increase NAD^+^ levels and mitochondrial copy number in skeletal muscle (Uddin et al., 2016). Exogenous NAM supplementation to 28-month-old rats for 5 weeks can improve the exercise ability and metabolic level by regulating the SIRT1-PGC-1α signaling pathway (Pajk et al., 2017). NR supplementation, unlike NMN or NAM, did not increase skeletal muscle NAD^+^ content or skeletal muscle functions but did downregulate the expression of genes involved in glycolysis, TCA cycle and mitochondria biogenesis, both in old mice and elderly human (Yang et al., 2020). Despite this, NR supplementation reduced macrophage infiltration, demonstrating potent anti-inflammatory effects in aged mice muscle (Elhassan et al., 2019). Although the rational for the difference between NR and NMN remains unclear, these studies demonstrate that NAD^+^ precursors have therapeutic potentials for anti-inflammatory treatment or sarcopenia in old muscle.

#### Extracellular NAD^+^: Carry Out Inter-Tissue Communications

Although NAD^+^ is widely utilized within cells, it actually hardly diffuses through cell membrane (van Roermund et al., 1995). This is in line with the highly variant concentrations of NAD^+^ in different tissues. The current intervention strategy for NAD^+^ is to supplement membrane-permissible NAD^+^ intermediate NMN or NR to manipulate NAD^+^ production in cell (Yoshino et al., 2018). Is NAD^+^ capable to influence across tissues? The answer is “yes.” A secreted form of NAD^+^ synthesis enzyme eNAMPT (also named as PBEF or Visfatin), secreted by adipose tissue, can increase NAD^+^ content in hypothalamus, hippocampus, pancreas, or retina and delay aging in mice (Yoon et al., 2015; Yoshida et al., 2019). However, it is also reported to have some malignant functions, for example, to induce endothelial dysfunction by activating NLRP3 inflammasome and facilitate release of IL-1β (Romacho et al., 2020) or to amplify preclinical acute lung injury (Quijada et al., 2020), or to cause mouse and human beta cell dysfunction in type 2 diabetes at high concentrations (Sayers et al., 2020). Given its flexible nature of being a cytokine, eNAMPT has a potential to impact across tissues and channel NAD^+^ metabolism systematically in human body, which makes it a promising intervention target for the future research.

Extracellular NAD^+^ itself can be signaling messengers. It was reported to elevate cytosol calcium signaling and promote apoptosis in the cultured human osteoblastic cells (Romanello et al., 2001). CD38 and CD157, existing on the outer membrane of cell, catalyze the cleavage of extracellular NAD^+^ into ADP-ribose or cyclic ADP-ribose, which are responsible for activating immune function and induce antibody response (Partida-Sánchez et al., 2004). In addition, extracellular NAD^+^ induce apoptosis in mice T cells but not B cells, possibly due to the cleavage by another membrane protein mono-ADP-ribosyl transferases (ARTs) (Seman et al., 2003). In summary, although extracellular NAD^+^ barely diffuse into cytosol, itself or derived molecules from its catabolism can transduce signals into cell to induce immune responses cascade. Since most of the extracellular NAD^+^ molecules probably come from the broken cells as a result of stimulus or stress, this makes it a suitable “sensor” to initiate self-defense mechanisms such as immune clearance. The research on functions of extracellular NAD^+^ has been long overlooked which deserves a deeper investigation in the future.

### Circulating Protein Factors in Blood and Their Anti-aging Effects

The suspicion that blood carries anti-aging factors has a long history traced back to the rumors that vampires stay young by drinking human blood. A number of studies using parabiosis technique to channel blood streams of young and old mice had supported the prediction that certain factors related to aging circulate in the blood and manipulating their levels may have anti-aging effects (Finerty, 1952; Wright et al., 2001; Conboy et al., 2005). The parabiotic pairings between old and young mice enhanced the proliferation of skeletal muscle stem cells in the old mice by activating Notch signaling pathway and regenerating the aged hepatocytes by restoring cEBP-α complex (Conboy et al., 2005). Exposing old mice to the blood of the young parabiotic partner restored remyelination capacity in the central nervous system of old mice to produce new myelin sheaths by recruiting blood-derived monocytes from young mice (Ruckh et al., 2012). The old mice sharing blood stream with young mice for 4 weeks displayed dramatical improvement in their cardiovascular system including regressed cardiac hypertrophy, reduced cardiomyocyte size and remodeled molecular events. In this study, they used proteomics analysis to successfully identify the TGFβ superfamily member GDF11 is the circulating factor in young mice that improved the cardiac hypertrophy of old mice (Loffredo et al., 2013). However, another study later reported the contradict finding that GDF11 increased with age and repressed muscle regeneration (Egerman et al., 2015; Schafer et al., 2016). The anti-aging function of circulating GDF11 is still currently under debate.

Despite of the controversy about GDF11, researchers continue to search for factors critical for aging progress in the blood. A recent study found that prostaglandin-degrading enzyme 15-PGDH elevated in aged mice and contributed to sarcopenia. Pharmaceutically inhibition of 15-PGDH increased muscle mass and strength in old mice by increasing PGE2 levels, also improved mitochondrial function and autophagy by decreasing TGF-beta and ubiquitin- proteasome pathways (Palla et al., 2021). Another new finding reported that in aging macrophages and microglia, the lipid messenger prostaglandin E2 (PGE2), a major circulating modulator of inflammation, could reduce glucose flux and mitochondrial respiration through its EP2 receptor. The inhibition of myeloid EP2 sufficiently to restore cognition in aged mice by rejuvenating cellular bioenergetics, decreasing brain inflammation, and increasing hippocampal synaptic plasticity along with spatial memory (Minhas et al., 2021). The thrombospondin-4 (THBS4) and SPARC-like protein 1 (SPARCL1) that are enriched in young mice’s serum could also rejuvenate brain functions by promoting synapse formation and NMDA receptor recruitment (Gan and Sudhof, 2019). These studies prove that certain proteins or messenger molecules in the blood indeed involve in regulating metabolisms in tissues, which reassure the necessity to keep on searching for circulating factors with anti-aging effects.

### RNA Molecules and Their Roles in Shaping Aging Microenvironment

Besides proteins, cell-free nucleic acid molecules are emerging candidates of the beneficial factors for aging that attract relatively less research attentions. A large number of secreted circulating non-coding RNAs (ncRNAs) were found to influence senescence and the outcomes of age-related diseases by modulating microenvironment. For example, LncRNA-ANRIL inhibited cellular senescence of VSMCs by up-regulating miR-181a and down-regulating SIRT1 (Tan et al., 2019). LncRNA-NEAT1 promoted autophagy in the mice with MPTP-induced Parkinson’s disease by stabilizing PINK1 protein and reducing miR-374c-5p (Yan et al., 2018; Dong et al., 2021). In addition, NEAT1 accelerated the progression of liver fibrosis and hepatocellular carcinoma (HCCs) (Bu et al., 2020). In rheumatoid arthritis, secretion of LncRNA-HOTAIR is elevated to promote dissolution of bone and cartilage matrix, and the level of LncRNA-HOTAIR in serum can be used as a novel non-invasive biomarker for the diagnosis of rheumatoid arthritis (Song et al., 2015). In osteoarthritis, mesenchymal stem cells (MSCs) secreted KLF3-AS1 to inhibit apoptosis and promote proliferation of chondrocytes, resulting in increased secretion of miR-372-3p that promotes apoptosis of MSCs (Liu et al., 2018). In muscle, miR-199-3p was reported to enhance myogenic differentiation and muscle regeneration. Its decline with aging results in muscle fiber atrophy and loss of muscle strength, which can be rescued by administering miR-199-3p mimics (Fukuoka et al., 2021). As matter of fact, unlike proteins, circulating RNA’s effects on aging are, mostly tissue/organ specific and vary depending on the types of the source or recipient cells. One of such examples is LncRNA-MALAT1. Endothelial progenitor cells secreted LncRNA-MALAT1 to inactivate miR-124/ITGB1 pathway and promote migration of bone marrow-derived macrophage and differentiation of osteoclast precursors (Cui et al., 2019). But in atherosclerosis, LncRNA-MALAT1 secreted by HUVECs promotes M2 macrophage polarization via NF-κB/TNF-α pathway (Huang et al., 2018). Interestingly, a recent research studied the miRNA profile of the NMN-treated old mice that had shown significant functional vascular rejuvenation. The authors found that dozens of miRNAs up-regulated by NMN might contribute to the anti-aging changes in vascular function through anti-atherogenic effects and epigenetic rejuvenation (Kiss et al., 2019b). This study established a link between miRNAs and other major aging regulating factors, implying possible interplay.

Circular RNAs (circRNAs) are a new type of non-coding RNA molecules that regulates age-related diseases. The circRNAs are generated by back-splicing, which connects the downstream 5’ splice donor site with upstream 3’ the recipient site on the mRNA transcript, forming covalently closed loop structure (Starke et al., 2015). These circRNAs are specifically expressed during aging course of nervous system, muscle, skin, and visual system (Cai et al., 2019). Various microarray or RNA-seq studies suggested that fibrosis is often accompanied with the reduction or accumulation of different circRNAs (Li et al., 2018), which contribute to the pathogenesis. For example, based on the study on 306 blood samples of elderly people from the InCHIANTI study, circDEF6, circEP300, circFOXO3 and circFNDC3B were demonstrated to be age-associated (Haque et al., 2020). CircFOXO3 could combine with proteins including anti-senescence protein ID-1, transcription factor E2F1, Focal Adhesion Kinase (FAK) and HIF1α, to promote cardiac senescence (Du et al., 2016a). CircFoxo3 could also sequester cyclin CDK2 and P21 to prevent CDK2 from binding cyclin E, blocking cell cycle progression in the G1 phase (Du et al., 2016b). Silencing circFoxo3 could alleviate doxorubicin-induced cardiomyopathy (Du et al., 2016a). These findings suggest RNAs may act as a new biological marker of aging and shed light on new potential targets for anti-aging therapy.

Exploring the circulating aging-related factors in blood or in-between tissues has been a hot spot ever since modern aging research began. The attraction about this concept is that it is easier to be translated into anti-aging interventions and should have more systematically effects given the fact that blood basically can reach all the tissues. A number of therapeutic strategies interfering with circulating factors have proven anti-aging effects in brain, muscle or cardiovascular functions. So far these studies only looked into a certain type of tissue or organ. It is not clear whether rejuvenating multiple tissues could be achieved by a single circulating factor or multiple ones. It is a necessary question for scientists to solve in the future.

## Conclusion and Future Direction

In summary, we discussed the major mechanisms that could systematically change physiological functions at intercellular level and reshape microenvironment toward aging direction. Senescence and its secretion phenotype SASP are the most fundamental player here. They directly change compositions of cell population by arresting the proliferation of progenitor cells or release pro-inflammatory factors to chronically elevate basal inflammation level causing systematic inflammaging. The effects of senescence and SASP are “erosive.” Once it starts, it has the potential to spread via the flowing cytokines to induce secondary senescence in the remote. Elimination of senescent cells by senolytic drugs has been proven to be effective to counteract senescence in natural aging or age-related disease model (Kirkland and Tchkonia, 2017; Ge et al., 2021). Recently, the first clinical trial of senolytic drug was conducted in human with IPF. Surprisingly, instead of rescuing lung functions, there was significant improvement in locomotors function such as walking distance or gait speed (Justice et al., 2019). Although it is a mystery why the drug failed to take effect in lungs where the most of senescent cells existing in IPF patients, it is still exciting to see the improvement in motor functions which proved senescence communicate at inter-tissue levels. In the future, increasing the specificities of senolytic drug might help to better cure aging-related diseases. In the meantime, techniques to monitor or trace the primary senescence onset spot could also be helpful to constrain senescence at earliest stage as possible to avoid further damages.

In contrast to senescence and SASP, NAD^+^ and circulating anti-aging factors are mostly the mechanisms to counteract aging progression. The impacts of NAD^+^ supplementation on intracellular biological functions have been extensively studied, while the systematic effects of NAD^+^ at intercellular level still need a better understanding. When administrating NAD^+^ precursors NMN or NR, NAD^+^ concentrations do not increase at same levels in different tissues due to different accessibility or expression levels of NAD^+^ synthesis enzymes, which inevitably lead to different responses. The discovery of eNAMPT enzyme indicates that there exist inter-tissue communications to control or compensate the discrepancy of NAD^+^ production between tissues. Extracellular NAD^+^ metabolism remains a mystery yet deserves more research attentions. There are solid evidence supporting young blood can rejuvenate the physiology functions of old mice, proving the existence of certain circulating factors in the young mice that could help the old. Future study should keep making efforts to investigate these possible candidates. Blood has complicated composition. The currently proved aging-related circulating factors are not only proteins but also microRNAs. It should be kept in open mind that any component in blood, for example metabolite or hormone, could be aging-related. It is also possible that not a single factor but a combination of them involved. Regardless, more efforts still need to be made in searching for anti-aging factors in blood.

## Author Contributions

QT and XZ were responsible for writing Part 1. NL and JL were responsible for writing Part 2. QT designed the figures with the images modified from Servier Medical Art (Servier, www.smart.servier.com, licensed under a Creative Commons Attribution 3.0 Unported Licence). All the authors contributed to the article and approved the submitted version.

## Conflict of Interest

The authors declare that the research was conducted in the absence of any commercial or financial relationships that could be construed as a potential conflict of interest.
